# Molting in early Cambrian armored lobopodians

**DOI:** 10.1038/s42003-024-06440-x

**Published:** 2024-07-05

**Authors:** Ailin Chen, Jean Vannier, Jin Guo, Deng Wang, Piotr Gąsiorek, Jian Han, Wenjiao Ma

**Affiliations:** 1 Research Centre of Palaeobiology, Yuxi Normal University, 653100 Yuxi, China; 2 State Key Laboratory of Palaeobiology and Stratigraphy, Nanjing Institute of Geology and Palaeontology, Chinese Academy of Sciences, 210008 Nanjing, China; 3 Laboratoire de Géologie de Lyon: Terre, Planètes, Environnement (CNRS-UMR 5276), Université de Lyon, Université Claude Bernard Lyon 1, ENS de Lyon, CNRS, Villeurbanne, 69622 France; 4 Chengjiang Science Museum, Management Committee of the Chengjiang Fossil Site World Heritage, 652500 Chengjiang, China; 5 State Key Laboratory of Continental Dynamics, Shaanxi Key Laboratory of Early Life and Environments, Department of Geology, Northwest University, 710069 Xi’an, China; 6 Natural History Museum of Denmark, University of Copenhagen, Øster Voldgade 5-7, DK-1350, Universitetsparken 15, DK-2100 Copenhagen, Denmark; 7 Department of Invertebrate Evolution, Faculty of Biology, Jagiellonian University, Gronostajowa 9, 30-387 Kraków, Poland; 8 Yuxi Museum, 653100 Yuxi, China

**Keywords:** Palaeontology, Metabolism

## Abstract

Lobopodians represent a key step in the early history of ecdysozoans since they were the first animals to evolve legs within this clade. Their Cambrian representatives share a similar body plan with a typically cylindrical annulated trunk and a series of non-jointed legs. However, they do not form a monophyletic group and likely include ancestors of the three extant panarthropod lineages (Tardigrada, Onychophora, Euarthropoda). Some species display astonishing protective devices such as cuticular plates and spines. We describe here the armor and molting process of *Microdictyon* from the early Cambrian of China. *Microdictyon* secreted ovoid paired cuticular sclerites that were duplicated in a non-synchronous way along the animal’s body. The reticulated pattern and cuticular architecture of these sclerites have similarities to extant armored tardigrades that recently served in hypothesizing that tardigrades are possibly miniaturized lobopodians. Ecdysis and hard cuticular protection are now well documented in the whole spectrum of early Cambrian ecdysozoans such as soft-bodied scalidophorans, lobopodians and fully articulated euarthropods. We hypothesize that the secretion of sclerotized cuticular elements periodically renewed via ecdysis was a key innovation that opened large-scale evolutionary opportunities to invertebrate animal life, specifically ecdysozoans, both in terms of anatomical functionalities and ecological success.

## Abstract (web summary)

This paper presents exceptionally preserved fossils from the early Cambrian that show how molting occurred in some of the very earliest arthropod relatives, a trait which enabled continuous growth in animals with hard exoskeletons.

## Introduction

Ecdysozoans form a huge animal clade^1^ that includes euarthropods (hexapods, crustaceans, myriapods, chelicerates), onychophorans, tardigrades, and a variety of scalidophoran (e.g. priapulids) and nematoid vermiform organisms, and have a very rich fossil record from the early Cambrian onwards. In contrast to all other animals that grow in a gradual manner, all ecdysozoans undergo stepwise molting stages during which their cuticle is shed and renewed. The intermolt stage is characterized by the expansion of internal tissues, whereas the external size of the exoskeleton remains unchanged, thus giving the ecdysozoan growth a unique incremental pattern, well-exemplified by modern euarthropods. Ecdysis in crustaceans and insects is controlled by a complex gene regulatory network, the endocrine system and specific neurosecretors, resulting in the biosynthesis of ecdysteroids such as ecdysone (E) and 20-hydroxyecdysone (20E) (e.g. refs. ^2–4^). The molting pathway of other ecdysozoan groups, noticeably the scalidophorans, is, however, far less understood. Comparative biochemical, genomic and transcriptomic analyses^3^ revealed that the required genes responsible for the biosynthesis of ecdysteroids are present in non-arthropod and even in non-ecdysozoan groups, suggesting that key genetic elements of the molting pathway probably evolved prior to the rise of arthropods (early Cambrian) and perhaps among some of the deepest branches of the animal tree (e.g. ecdysis-related neuropeptids^5^). Although fossil ecdysozoans can rarely be observed during the act of molting (e.g. Cambrian euarthropods such as *Marrella*^6^ and *Alacaris*^7^, loriciferans^8^ and worms such as *Cricocosmia*^9^), exuviae do occur in the fossil record. Several criteria can be used to confidently distinguish exoskeleton molts (exuviae) from carcasses (whole bodies), especially in trilobites^10,11^ that grew through incremental growth stages as modern arthropods. Large accumulations of assumed exuviae suggest that the mid-Cambrian Burgess Shale euarthropods *Alalcomenaeus* and *Canadaspis* performed synchronized molting^12^ as do extant crustaceans such as krill in which molting and mating occur simultaneously^13^. Recently, ecdysis has been described in 535-million-year-old scalidophorans from China^14^. These vermiform organisms molted in a manner similar to that of extant priapulids, i.e., by turning their old cuticle inside out or by extricating themselves smoothly from their exuvia. These fossil exuviae provide the oldest known direct evidence of ecdysis in animals and show that molting was already operational among one the most basal ecdysozoan groups (e.g. ref. ^15^), well before the divergence of panarthropods.

We focus here on Cambrian lobopodians that, in most recent phylogenies (e.g. ref. ^16^), stand in a relatively basal position and represent the earliest leg-bearing animals, and describe their molting process in detail. Lobopodians are non-segmented panarthropods^17^ resembling modern onychophorans, characterized by soft legs and, in many taxa, by a paired dorsal armature made of rigid sclerites (e.g. *Microdictyon*) or spines with a cone-in-cone structure (e.g. *Hallucigenia*^18,19^). Their molting behavior has received little attention from scientists, although duplicated sclerites have been reported in Cambrian small shelly fossils (SSF) assemblages^20,21^ and Burgess-Shale-type (BST) Lagerstätten^22^, and interpreted as possibly resulting from the overlap of old and newly secreted sclerites. We describe here almost complete specimens of the lobopodian *Microdictyon sinicum* (see refs. ^22–24^) that were buried alive in sediment during the pre-molt stage. Although lobopodians have no direct counterpart among modern animals, their sclerite structure and segmental distribution resemble that of extant armored limno-terrestrial tardigrades, the echiniscids. We analyze these similarities for the first time in light of recent evolutionary scenarios^25^. Finally, the secretion of sclerotized elements coupled with ecdysis are seen as key innovations that might have shaped the early evolution of animal life and ecosystems.

## Results and discussion

### Description of fossil specimens

**Fig. 1:**
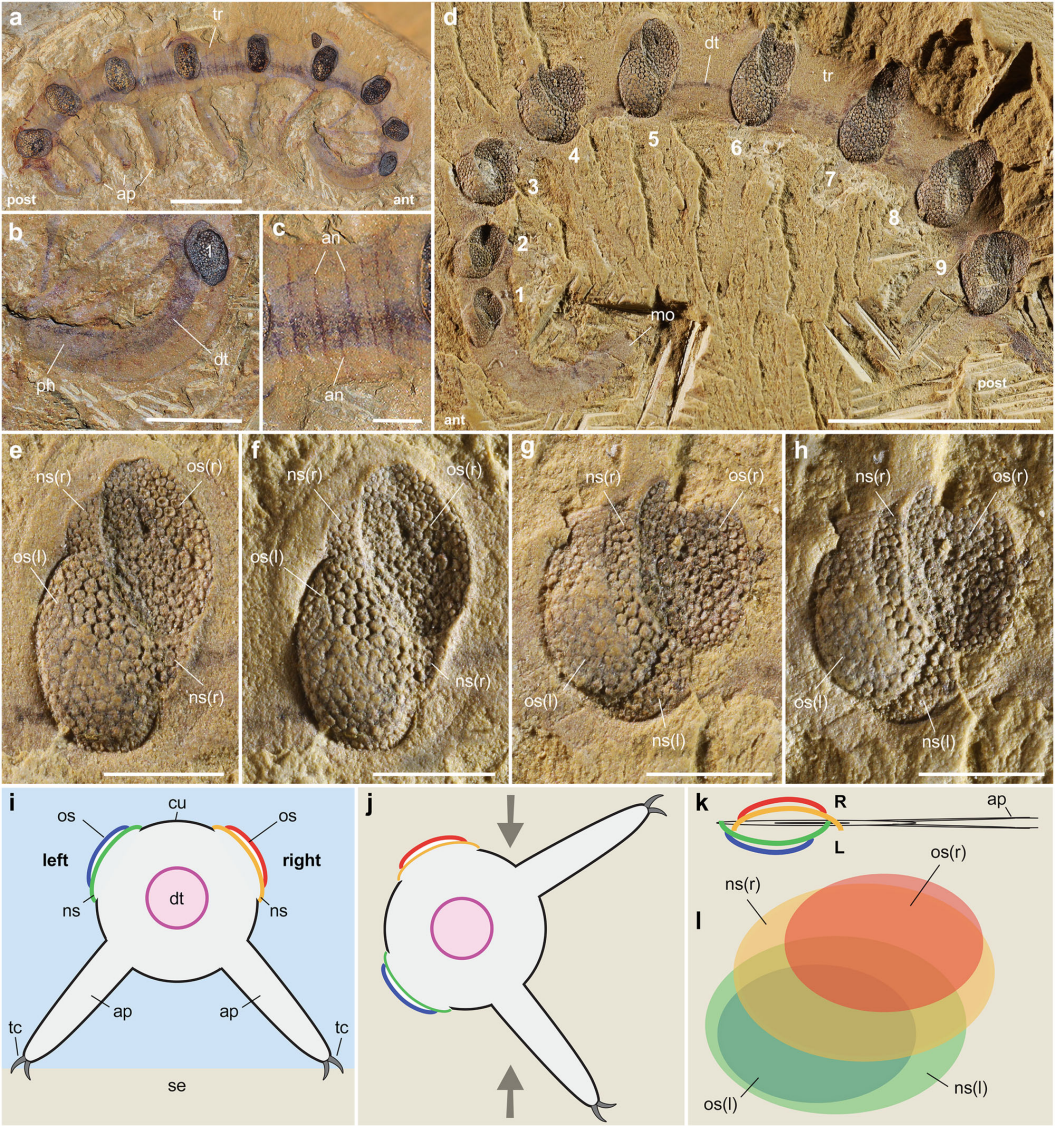
Molting in *Microdictyon sinicum* from the early Cambrian Chengjiang Lagerstätte. **a–c** General anatomy of the animal *Microdictyon*; complete specimen (YRCP0041, Jiucun section, Chengjiang City); lateral view, details of mouth and pharynx area, and trunk annulation, respectively. **d** General view of a laterally compressed specimen (YRCP0040, Xiaolantian section) showing duplicated sclerites (anterior part points to left). **e**, **f** Duplicated sclerites (5th pair; see location in **d**) from different angles of illumination. **g**, **h** Duplicated sclerites (4th pair; see location in **d**) from different angles of illumination. **i**, **j** Idealized diagram to show overlapping old and new sclerite on both sides of the body, in life attitude and after burial (body rotated ca. 90° anticlockwise); grey arrows indicate compaction in sediment. **k** Laterally compressed specimen showing right and left sets of sclerites. **l** Sclerite overlapping represented by colored areas (new sclerites larger than old ones). Sclerites are numbered 1–9 from anterior to posterior. an annulation, ANT anterior end, ap appendage, bo body, cu cuticle, dt digestive tract, L left body side, no node, ns new sclerite, ns(l) new sclerite (left), ns(r) new sclerite (right), nr negative relief; os old sclerite, os(l) old sclerite (left), os(r) old sclerite (right), POST posterior end, pr positive relief, R right body side, ri ring, se sediment, tc terminal claw, w wall of reticulum. Scales bars: 1 cm in (**a**); 5 mm in (**b**, **d**); 2 mm in (**c**); 1 mm in (**e–h**).

**Fig. 2:**
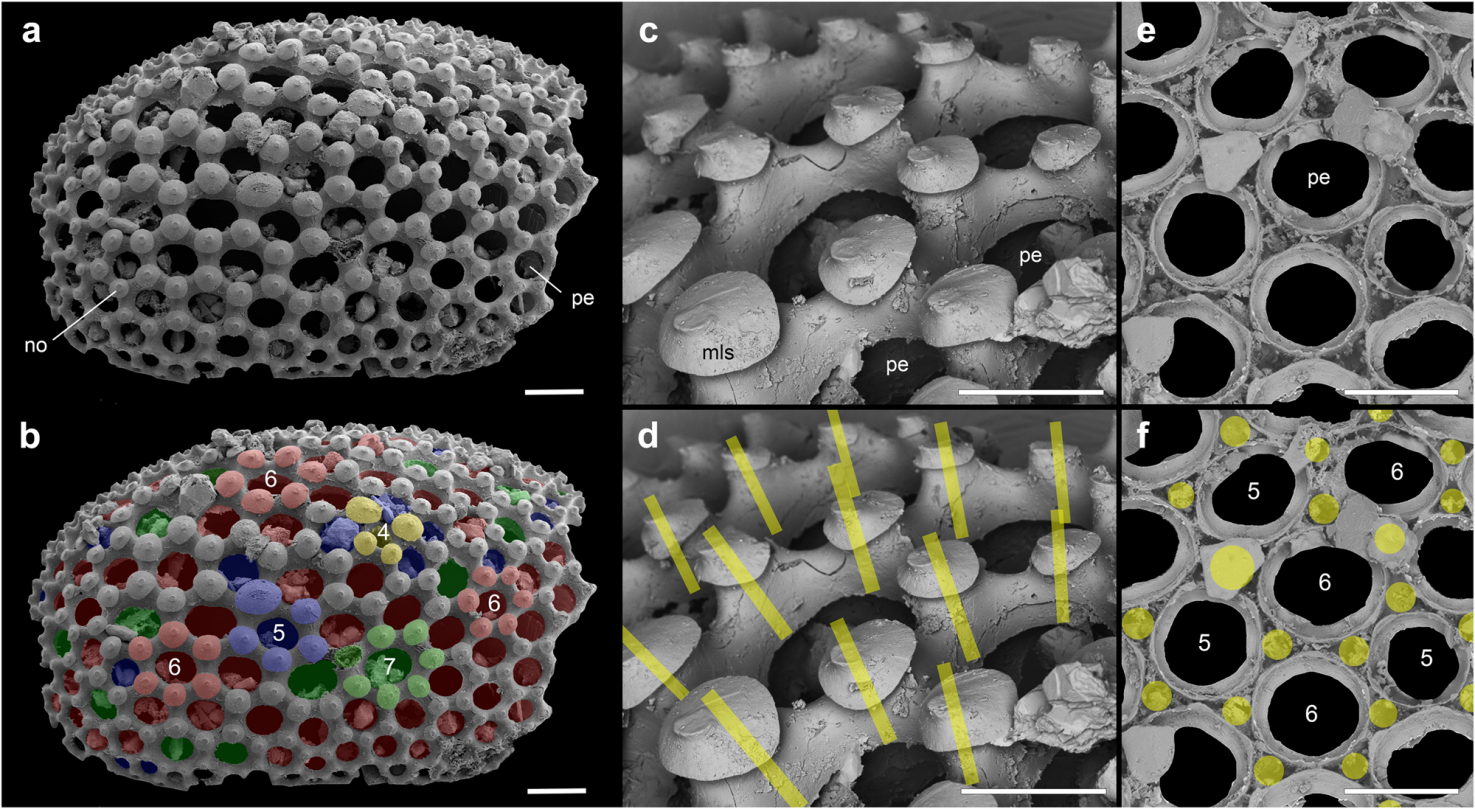
Secondarily phosphatized cuticular plates in *Microdictyon* sp. from the early Cambrian Xinji Formation, Shuiyu section, Shanxi Province, China (see ref. ^44^). **a**, **b** General view showing circular perforations and nodes with a polygonal distribution; quadra-, penta-, hexa- and heptagonal pattern highlighted in yellow, blue, red and green color; hexagonal pattern dominant. **c**, **d** Details of cuticular wall around perforations showing mushroom-like features at nodes; yellow lines indicate central axis of elevated features at nodes. **e, f** Details on the inner surface showing rimmed circular perforations; yellow circles indicate the basis of nodes. All SEM images, courtesy of Bing Pan. mls mushroom-like structure, no node, pe perforation, 4–7 quadragonal to heptagonal pattern. Scale bars: 200 µm in (**a**, **b**, **e**, **f**); 100 µm in (**c**, **d**).

The fossil specimens described here have the diagnostic features of *Microdictyon sinicum* (e.g. refs. ^26,27^) such as a tubular body tapering anteriorly into a limbless curved projection, nine pairs of ovoid trunk sclerites bearing a hexagonal mesh, cylindrical perforations and spiky nodes (Figs. 1a–h, 2; Supplementary Fig. 1). Each pair of sclerites is inserted in line with a pair of slender legs bearing terminal claws (Fig. 1i, j). The limbless extension of *Microdictyon* most probably represents the anterior part of the animal, based on comparisons with other lobopodians (see ref. ^28^ for homologies), such as *Hallucigenia* in which eyes and pharynx are accommodated within a comparable anterior feature^18,19^. The number of appendages is likely to be nine pairs^26^ rather than ten^27^. The terminal pair of limbs is the shortest one and its right and left counterparts often lie within the same plane, thus creating the false impression of two distinct pairs of appendages (see ref. ^27^, Fig. 18). The sclerites of *Microdictyon* were most probably organic, possibly chitinous, as are the rigid spikes of *Hallucigenia*^18^.

**Fig. 3:**
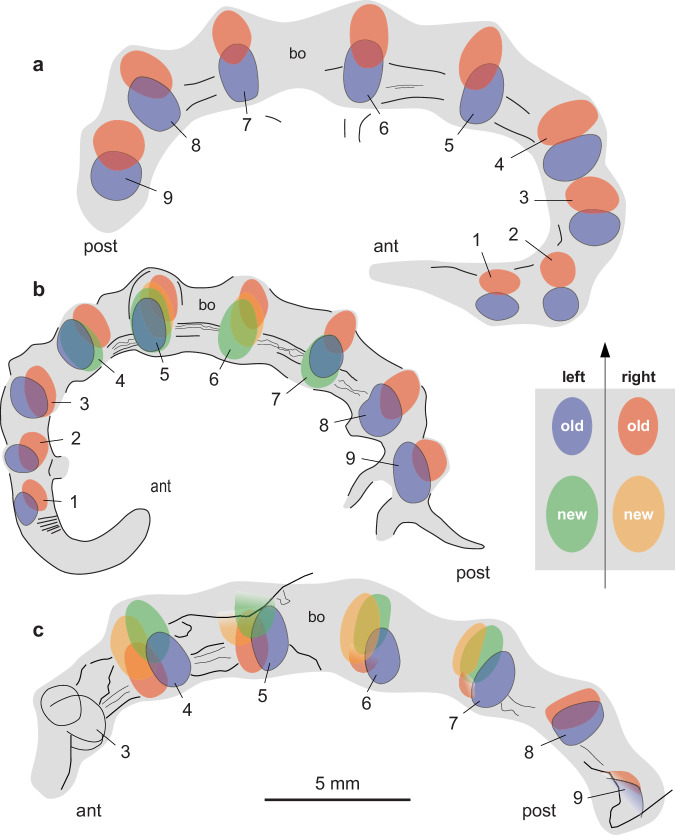
Sclerite arrangement in *Microdictyon sinicum* from the early Cambrian Chengjiang Lagerstätte. **a** Complete specimen at intermolt stage (CJHMD-MZ19007; see Fig. 2a, specimen 1); note sight overlap of right and left sclerites (1^st^–4^th^ pairs). **b** Complete specimen at molting stage (YRCP0040; see Fig. 1d) showing sclerite duplication in the middle part of the body. **c** Incomplete specimen at molting stage (CJHMD-MZ19007; see Fig. 4a, specimen 2 and Fig. 4b) showing sclerite duplication in the middle part of the body. Sclerites are numbered 1–9 from anterior (black arrow) to posterior. Body in light gray. Sclerites along the right body side are represented in red (old sclerite) and orange (new sclerite), and those along the left side in blue (old sclerite) and green (new sclerite); see explanation Fig. 1i–k. ant anterior end, bo body, post posterior end. Scale 5 mm for all specimens.

**Fig. 4:**
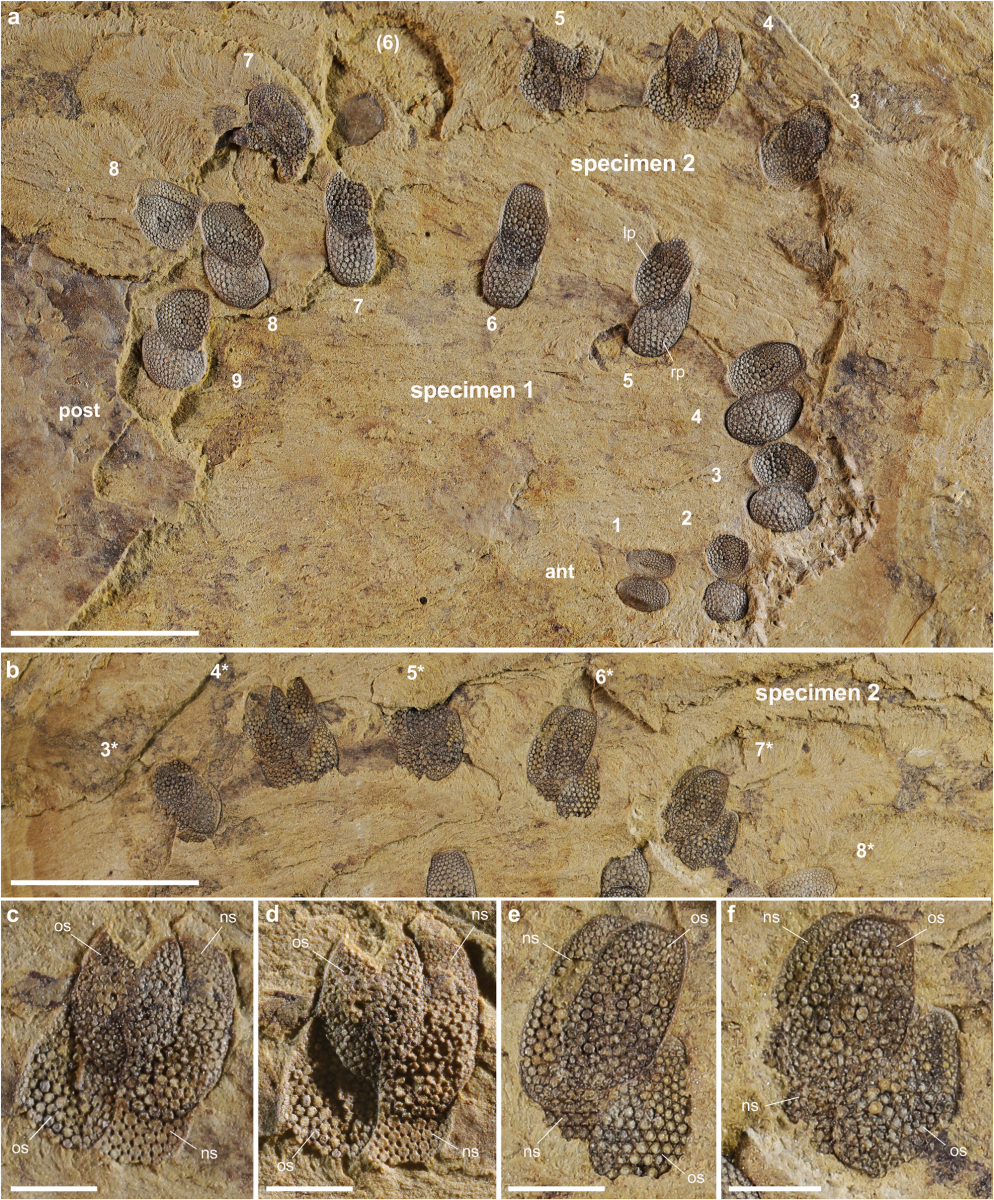
Molting in *Microdictyon sinicum* from the early Cambrian Chengjiang Lagerstätte. **a**, **b** General view of two laterally compressed specimens on the same slab (Jiucun section; CJHMD-MZ19007), part and counterpart (note that only one part of the rock slab is shown), respectively; specimen 1 with 9 pairs of sclerites, intermolt stage; specimen 2 with duplicated sclerites, molting stage. **c**, **d** Details of 5^th^ sclerites (see location in **a**, part) from different angles of illumination. **e**, **f** Detail of 3^rd^ and 2^nd^ sclerites, respectively (see location in **b**, counterpart). Sclerites are numbered 1–9 from anterior to posterior in specimen 1, tentatively 3–8 in specimen 2 (counterpart, 3*–8* in **b**), the anterior polarity being uncertain. ant anterior end, ns new sclerite, op old sclerite, post posterior end. Scale bars: 5 mm in (**a**, **b**); 1 mm in (**c–f**).

Specimen YRCP0040 (Xiaolantian section; Figs. 1d, 3b) displays a complete set of paired sclerites preserved in situ, some of them being duplicated. Pairs 5^th^–9^th^ show a slight overlap of the right sclerites over the left ones. This configuration results from the collapse of soft tissues after death, followed by lateral compression after burial in sediment, causing the four rigid, duplicated sclerites to come into contact with a slight offset. The lack of duplication in the 1^st^ and 2^nd^ pairs suggests that the molting process was not completely achieved when the animal died and was buried in the sediment. Specimen CJ-MZ19007(1) (Jiucun section; Figs. 3a, 4a) is well-preserved with nine series of sclerites distributed in pairs along the body. The 1^st^–3^rd^, 8^th^ and 9^th^ pairs show a moderate overlap of the left sclerite over the right one. Pairs 4^th^–7^th^ are duplicated, each with two sets of stalked sclerites. Each duplicated set consists of a larger elliptical element topped with a smaller one, i.e. that the new sclerite is larger than the old one (exuvia). Lateral compression resulted in bringing the two sets of sclerites into contact, the right and left one being made up of concave and convex stacked elements, respectively. Specimen CJ-MZ19007(1) is interpreted as being fossilized in the process of molting. CJ-MZ19007(2) (Jiucun section; Fig. 4a) is an incomplete specimen (anterior part missing) that similarly exhibits four pairs (4^th^–7^th^) of duplicated sclerites in the midpart of the body, each with a set of concave and convex elements either stacked or displaced (Fig. 4b–f). YMYD082 (Maotianshan section; Supplementary Fig. 1) is an additional incomplete specimen showing duplicated sclerites most distinctly on the third-to-fifth pairs.

### Molting process in lobopodians

The four specimens of *Microdictyon* described here confirm that lobopodians renewed their cuticle and grew through successive molting stages and reveal previously unknown details of their molting process. The duplicated right and left sets of sclerites suggest that *Microdictyon* did not shed off its old sclerites immediately after the new ones were formed and that new and old sclerites both remained associated for a while. Isolated sets of stacked sclerites found in other lobopodian species such as *Microdictyon jinshaense*, *M. chinense* and *Quadratopora zhenbaensis* among early Cambrian SSF assemblages^29^ similarly indicate the temporary retention of the old sclerite over the new one. The presence of both duplicated and non-duplicated sclerites along the same specimen (e.g. Fig. 3) suggests that ecdysis did not take place synchronously along the body but, instead, possibly started in the central region and then spread anteriorly and posteriorly. Interestingly, this recalls the biphasic molting seen in both terrestrial and marine isopods (e.g. refs. ^30,31^). In contrast with the vast majority of crustaceans that perform monophasic molting, these isopods renew their exoskeleton following two steps- i.e. its posterior part is shed before the anterior one due to the differential responses of the anterior and posterior part of the animal to ecdysteroids. The hypothetical asynchronous molting of *Microdictyon* might find its origin in comparable variations of the biochemical signal.

The molting mechanism of lobopodians, in its general principles, seems to have been comparable with that of other ecdysozoans (e.g. modern crustaceans). However, it applied to a heterogenous cuticular system made of rigid sclerites and a thinner flexible cuticle that lined the whole annulated body and appendages. We suggest that molting possibly took place through the following sequence (Supplementary Fig. 2): (1) At the intermolt stage the animal was fully protected by a thin and flexible cuticular layer secreted by underlying epidermal cells; this layer was locally thickened and sclerotized to form paired rigid elliptical sclerites; (2) Based on the molting process of extant ecdysozoans, we assume that the secretion of fluid between the epidermis and the cuticle (apolysis) resulted in lysing the basal part of the cuticle, thus initiating ecdysis; (3) Cell division occurred within the epidermal layer and new cuticular material was secreted, including new sclerites; however, old and new sclerites were not completely dissociated from each other at that stage; (4) The final step was characterized by the breakup of the old cuticle and the release of the exuvia whereas the newly secreted sclerites kept growing thicker and possibly harder (sclerotization). The hypothesis that molting may have initiated in the central part of the body would suggest that the animal pulled itself out through a possible mid-dorsal split (Supplementary Fig. 3) and via repeated muscular contractions, as observed in extant crustaceans (e.g. ref. ^32^) and insects, keeping in mind that a great variety of molting mechanisms occurs among both groups. This mechanical process would have facilitated the shedding of the thin and relatively long ventral appendages. As in the vast majority of present-day euarthropods, *Alacaris mirabilis*, an early Cambrian fuxianhuiid^7^ molted its exoskeleton dorsally through a large gap that appeared at the base of its head shield. *Microdictyon* is probably no exception to the rule, with a newly molted animal emerging dorsally and not ventrally, as assumed for the lobopodian *Xenusion*^33^.

The duration of the whole molting process (premolt, ecdysis, postmolt) of extant euarthropods varies according to species and environmental conditions, from several days to months^34^. However, the active phase of shedding, i.e. when the animal extricates itself from its old cuticle, is relatively short (15 minutes in crayfish^32^). The duration of the whole molting process is impossible to estimate in *Microdictyon*. What we observe in the case of *Microdictyon* corresponds to the pre-molt stage preceding ecdysis.

### Composition and function of lobopodian sclerites

Lobopodian sclerites often occur in small shelly fossils (SSF) assemblages as secondarily phosphatized isolated elements, but no evidence points to an originally biomineralized composition. Secondary phosphatization affects both fully organic and biomineralized (e.g. calcified) tissues. For example, the grasping spines of Cambrian chaetognaths, although mineralized in calcium phosphate in SSF assemblages, show an organic composition in Burgess-Shale-type Lagerstätten (see ref. ^35^) and are chitinous in modern species^36,37^. Similarly, Steiner et al.^38^ showed that the sclerites of *Microdictyon sinicum*, *Hallucigenia hongmeia*, *H. fortis* and *Onychodictyon ferox* are mainly enriched in Fe and C and devoid of detectable traces of Ca and P. These latter chemical elements may have been removed during diagenesis or, more likely, were originally absent. The dorsal spines of *Hallucigenia* from both BST localities and small carbonaceous fossils (SCF) have remarkably well-preserved microstructures such as internal nested construction and external ornamentation made of irregularly distributed microscales^18,19^. These spines show an overwhelmingly organic composition with only very faint traces of P in C-rich areas. Similarly, small amounts of P occur in the cuticle of modern arthropods (e.g. insects^39^ and isopods^40^) as nano-sized granules of amorphous calcium phosphate that do not constitute a mineralized layer *sensu stricto* but may simply enhance cuticular hardness. The diffused presence of P in lobopodian sclerites from BST-localities may result from a comparable original cuticular composition. We agree with other authors^38,41^ that the sclerites of all known Cambrian lobopodians were originally mainly organic, possibly chitinous, irrespective of their morphology (e.g. spines, cones, perforated sclerites). This, together with a consistent dorsal location in pairs aligned to appendages, supports the hypothesis^38^ that these various cuticular elements are all homologous.

The function of the *Microdictyon* sclerites has been subject to various interpretations (e.g. ref. ^29^). Chen et al.^42^ and Budd^43^ suggested that they served as anchoring areas for muscles. However, no potential attachment areas such as scars are visible along their internal surface (e.g. ref. ^44^; Fig. 2e, f) and rare lobopodians with well-preserved muscles^45^ show no oblique fibers attached dorsally to the inner wall of the cuticle. The hexagonal pattern of the *Microdictyon* sclerites has been compared with that of arthropod compound eyes and interpreted as structures possibly supporting eye lenses^46^. Multiple eyes are known in Cambrian arthropods (e.g. *Opabinia*^47,48^) but are concentrated in the head. As pointed out by Zhang et al.^29^, if *Microdictyon* had multiple eyes, then it would make it a unique case among lobopodians that in their great majority do not possess perforated sclerites (e.g. *Hallucigenia*). Above all, the strongest argument against this hypothesis is that true eyes occur in the head region of several lobopodians as tiny ocelli (e.g. *Miraluolishania, Hallucigenia* and *Cardiodictyon*; see refs. ^49–52^). A protective function remains by far the most plausible option^28^. All types of dorsal sclerites, *a fortiori* the pointed ones (e.g. *Hallucigenia*) might have had a defensive or deterrent function at least against potential predators of comparable size.

### Comparisons with Cambrian worms

Yu et al.^9^ described the molting process of *Cricocosmia jinningensis*, a palaeoscolecidomorph worm that is particularly abundant in the early Cambrian Chengjiang localities. Before complete ecdysis, this worm displayed duplicated longitudinal rows of dorsal sclerites that clearly indicate an on-going splitting process between old and new cuticle identical in every way to that seen in *Microdictyon*. Whether the sclerites of *Cricocosmia* are homologous to those of *Microdictyon* remains uncertain. However, one could not exclude that the genes involved in the dorsal, serial and symmetrical location and secretion of these trunk sclerites were similar in both ecdysozoan groups. How *Cricoscomia* got rid of its old cuticle via an assumed ecdysial break between the introvert and the trunk as in modern priapulids^14^, or in a different way, remains unclear^9^. However, its lack of paired legs and the extremely elongated muscular trunk would suggest that the ecdysial process of palaeoscolecidomorph worms was different from that of lobopodians.

### Similarities with the cuticular plates of modern tardigrades

**Fig. 5:**
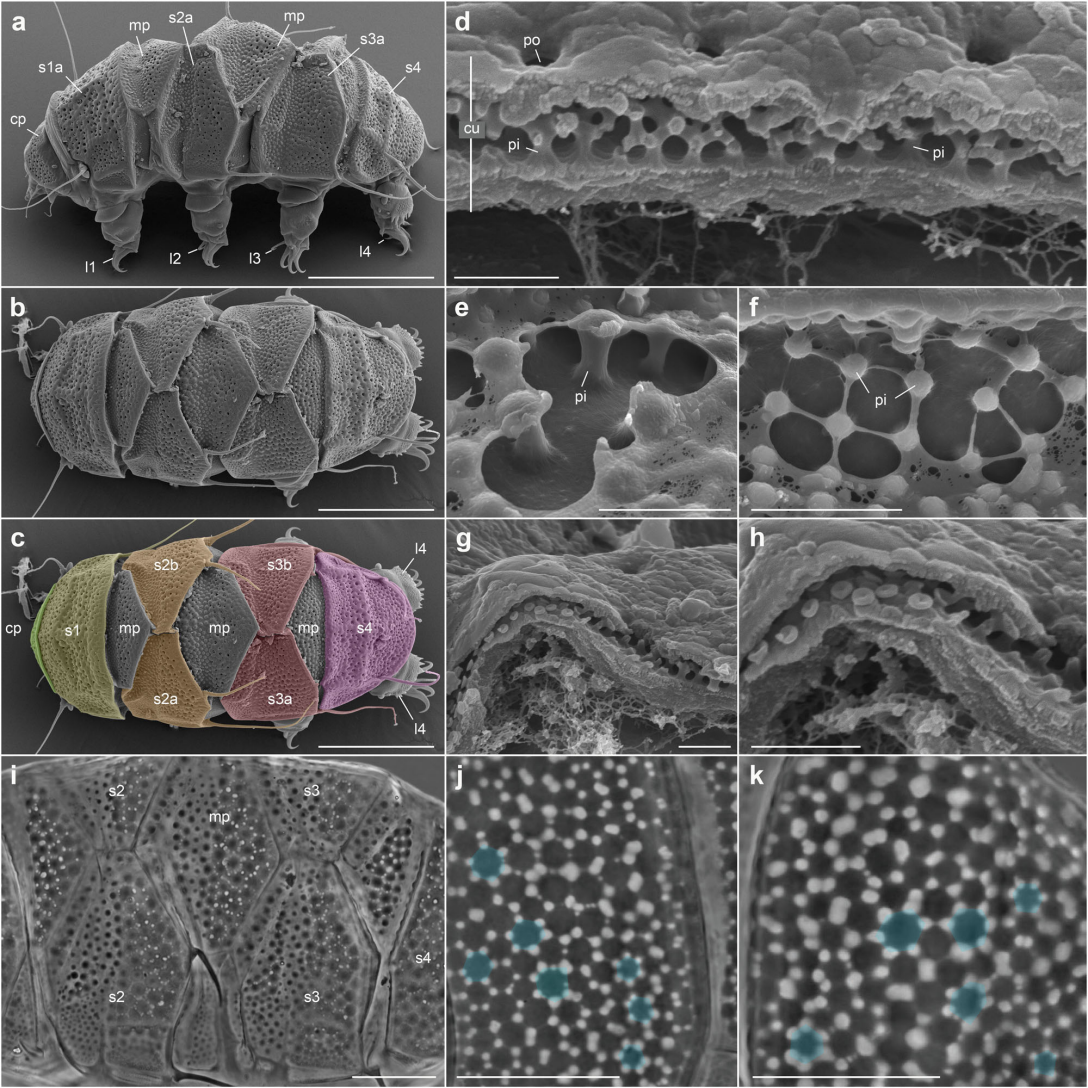
Cuticular plates in extant armored tardigrades (Heterotardigrada, Echiniscidae). **a–c** General morphology of *Echiniscus pellucidus* in lateral and dorsal views (cephalic and segmental plates highlighted in colors). **d**, **g**, **h** Transverse sections through a plate of *Barbaria ganczareki* showing pillars and internal hollows within the cuticle. **e**, **f** Pillars in the cuticle of *Cornechiniscus madagascariensis*. **i** Intermediate dorsal view of *Echiniscus pellucidus* showing plates and internal hollows within the cuticle. **j**, **k** Details of plate cuticular structure in *Claxtonia mauccii * showing pillar-like epicuticular bumps distributed in polygons (blue). **a–h** are SEM images, **i–k** photographs in phase contrast microscopy. cp cephalic plate, cu cuticle, l1–l4 first to fourth pair of legs, mp median plate, pi pillar, s1–s4 segmental plates (s1 = scapular plate; s4 = caudal plate, s2 and s3 are paired plates with **a** and **b** being the left and right elements, respectively) Scale bars: 50 µm in **a–c**; 20 µm in **i–k**; 5 µm in **e**, **f**; 1 µm in **d**, **g**, **h**. **d**, **g**, **h**, courtesy of Łukasz Michalczyk (see also refs. ^56–63,76^).

Tardigrades are microscopic panarthropods with four pairs of legs ending in well-developed claws, or more rarely, suction disks and pads in marine forms^53,54^, many species living in mosses and lichens. The cuticle of most limno-terrestrial heterotardigrades is locally thickened and forms an armor made of dorsal and dorsolateral sclerotized elements such as the cephalic, segmental and median plates that all display bilateral symmetry (Fig. 5a–c; see examples in refs. ^55–58^). Although non-ovoid, these paired segmental plates aligned with appendages (Fig. 5a) recall those of *Microdictyon* (Fig. 1a; Supplementary Fig. 1a, b), Other similarities may concern the structure of the cuticle itself. The cuticle of heterotardigrades is complex (Fig. 5d–k)^59–61^ and exhibits important variations both in external appearance and ultrastructure (e.g. pores, pseudopores, “spongy” layer^59^). One of the most original features of the heterotardigrade cuticle and particularly evident in dorsal plates, is probably the vertical pillars that link the lower part of the procuticle to the most external layer, the outer epicuticle (Fig. 5d, e; see refs. ^56,62^). These pillars may interconnect via thin bridges called striae or a more extensive layer (Fig. 5e, f), and outer elements of epicuticle are often distributed to form more or less regular polygonal patterns (e.g. hexagonal; Fig. 5j, k). This pillar structure creates a complex network of internal hollows within the cuticular plates that is either closed (“spongy” structure) or open to the exterior (e.g. via pores^63^). Interestingly, the sclerites of *Microdictyon*, although ca. 25 times larger (width), share important features with those of tardigrades, such as (1) a polygonal pattern (4- to 7-, hexagonal dominant; compare Figs. 2b and 5j, k; see ref. ^63^) with spiky or mushroom-shaped nodes^44^ (Fig. 2c, d) reminiscent of the pillar structure (Fig. 5d–h) seen in tardigrades; (2) a cuticular architecture made of circular perforations (Fig. 2) and thin walls that correspond to the hollow structure of the tardigrade cuticle (Fig. 5d). The similarities between lobopodians and tardigrades, although rarely explored^25^, go far beyond their plates and concern other key aspects of their body plan such as their soft (ancestrally telescopic) legs terminated with claws, often terminal mouth, pharyngeal structure and the lack of strong head differentiation^25^. Thus, close phylogenetic relationships between tardigrades and luolishaniid lobopodians have recently been hypothesized^25^, suggesting that tardigrades and luolishaniid lobopodians share a common ancestor, echoing earlier studies (e.g. *Antennacanthopodia*^64^, *Onychodictyon ferox*^19^, and *Aysheaia*^65^). Kihm et al.^25^ suggested that luolishaniids, a group of Cambrian lobopodians without sclerites, had sister-relationships with Tardigrada, thus challenging evolutionary models that instead favor relations with onychophorans^19,52^. In their phylogenetic tree, luolishaniid lobopodians appear as a possible stem-group Tardigrada, based on characters shared with modern tardigrades (differentiation of legs into two types, dorso-lateral paired structures on mid-head). *Luolishania* has a rounded head separated from the trunk by a slight constriction. The trunk bears 16 annulated, relatively long legs, each being seemingly associated with a pair of rounded bumps interpreted as sclerites^26,27^. Surprisingly, other lobopodians bearing sclerites, such as *Microdictyon, Hallucigenia, Cardiodictyon* and *Onychodictyon* were resolved in a more basal position before the divergence of Euarthropoda and Onychophora. Although a new phylogenetic study is out of the scope of our paper, Kihm et al.’s phylogeny^25^ raises questions. The presence of paired sclerites in *Microdictyon* and allied forms, comparable to those of modern tardigrades, appears as a new character (not considered in Kihm et al.^25^), and is either a convergence given that the echiniscoideans are a highly specialized heterotardigrade clade adapted to limno-terrestrial environments or, alternatively, might be interpreted as plesiomorphic. Sclerotized dorsal and ventral plates also occur in marine heterotardigrades that are considered as retaining ancestral tardigrade morphology. If so, this character may be of potential significance to resolve the ancestry of tardigrades and, more generally, the placement of lobopodians along the three branches of the panarthropod tree. The detailed description of ca. 250–350 µm long tardigrade-like microfossils from a Middle Cambrian Orsten-type locality in Siberia^66,67^ may provide new information on the anatomy of early ecdysozoans and how they differ or not from modern representatives of Tardigrada (Gąsiorek and collaborators, in-progress).

### The rise and evolutionary importance of cuticular protection

Direct evidence for molting is now available for many ecdysozoan groups that co-existed in the early Cambrian such as scalidophorans^9,14^, lobopodians^23^ and euarthropods^10,11^. Body and trace fossils suggest that worm-like animals (ecdysozoan or not) lived in the Ediacaran (e.g. *Ikaria*^68^) and were the potential makers of numerous sub-horizontal trails and shallow burrows. *Treptichnus*^69,70^ is a widespread complex burrow system still used as a marker to define the Precambrian–Cambrian transition. Its detailed morphology and experimental ichnology^69^ using extant priapulids (*Priapulus*) both suggest that its maker was a tubular, annulated and contractible worm with an introvert, scalid rows and terminal mouth, thus closely resembling scalidophoran worms. Although the nature of the last common ancestor of ecdysozoans remains hypothetical^15^, the secretion of a cuticle may be seen as a key innovation that preceded or was contemporaneous with the radiation of ecdysozoans. Schmidt-Rhaesa^71^ suggested that a preexisted matrix (e.g. glycocalyx; glycoproteins linked to polysaccharide chains) secreted by epidermal cells served as a matrix for the deposition of other molecules such as collagen or chitin, thus giving rise to a cuticular layer. Non-ecdysozoan animals have no chitinous cuticle and use other various methods such as mucous (e.g. flatworms) or more resistant secretions (e.g. annelids) to create a protective boundary layer around their body.

We suggest that three major successive steps may have taken place during the Precambrian–Cambrian transition: (1) Cuticular secretion evolved from precursors with a ciliated epidermis and a glycocalyx; (2) Molting lifted the mechanical constraints imposed by the non-extensible cuticle and incompatible with continuous body growth, i.e. natural selection selected ecdysis as a successful option that reconciled protection and optimal body growth; (3) Sclerotization in the form of rigid cuticular sclerites evolved among vermiform organisms into a variety of complex cuticular 3D-structures such as conical or plate-like sclerites (e.g. palaeoscolecids^9,72,73^). Whereas the cuticle is an efficient physical barrier that prevents damage due to friction with sediment, sclerites provide additional protection and increase anchoring to sediment. Indeed, sclerotization is likely to have promoted burrowing lifestyles (see evidence from trace fossils; e.g. refs. ^69,70,74^).

Lobopodians not only inherited the capacity of molting from their probable vermiform legless ancestors but also that of secreting paired sclerites (e.g. spines or plates as in *Microdictyon*). Whereas sclerites were crucial to the early bioturbators of sediment, they probably played an equally important role for the pioneer epibenthic walkers, that of protecting their soft body dorsally against predators and physical damage. The crucial role of cuticular secretion/sclerotization associated with molting is even more evident in euarthropods that evolved more sophisticated articulated systems involving joints and muscles such as those already seen in early Cambrian megacheirans (arthropods with a great appendage; see, e.g. ref. ^16^) and more advanced forms. The success of euarthropods shown by the remarkable increase of their diversity and ecological expansion at the beginning of the Cambrian era, would not have been possible without the major innovations of molting and sclerite secretion that preceded their radiation.

## Methods

All studied fossil specimens were collected from the Xiaolantian, Jiucun, and Maotianshan sections, ca. 8 km from Chengjiang, Yunnan Province, China, that all belong to the Yu’anshan Member of the Qiongzhusi Formation, and correspond to the *Eoredlichia-Wutingaspis* biozone, Cambrian Stage 3. Microscopic observations and light photography of fossil specimens were made with a Leica M205C stereomicroscope and a Canon EOS 5D Mark IV digital camera. Details on the methods (scanning electron microscopy, SEM) used for imaging *Microdictyon* (Fig. 2) are in Pan et al.^44^. All fossil specimens are housed in the collections of the following Chinese institutions: Yuxi Normal University Research Center of Paleobiology (YRCP numbers), Yuxi, Yunnan Province; Chengjiang Science Museum of the Management Committee of the Chengjiang Fossil Site World Heritage (CJHMD-MZ numbers), Yunnan Province; Yuxi Museum (YM-YD numbers), Yuxi, Yunnan Province. Tardigrades prepared for SEM were first fixed in ethanol and then dehydrated in an ethanol/acetone series prior to critical point drying in liquid CO_2_. Dried specimens were mounted on stubs, coated with gold, and later observed in SEM (for more details see ref. ^75^). Information concerning the fossil material and extant tardigrades can be obtained from Ailin Chen (ailinchen@yxnu.edu.cn) and Piotr Gąsiorek (piotr.lukas.gasiorek@gmail.com), respectively.

### Supplementary information

Supplementary Information
