# Supplementary material for: Molting in early Cambrian armored lobopodians
Source: Commun Biol. 2024 Jul 5;7:820. doi: 10.1038/s42003-024-06440-x (PMC11226638; doi:10.1038/s42003-024-06440-x)
Supplement: Supplementary file 1 — Supplementary Information [file 42003_2024_6440_MOESM1_ESM.pdf]

Molting in early Cambrian armoured lobopodians

Ailin Chen, Jean Vannier, Jin Guo, Deng Wang, Piotr Gąsiorek, Jian Han, and Wenjiao Ma

SUPPLEMENTARY INFORMATION

Supplementary Figure 1. Molting in *Microdictyon sinicum* from the early Cambrian Chengjiang Lagerstätte.

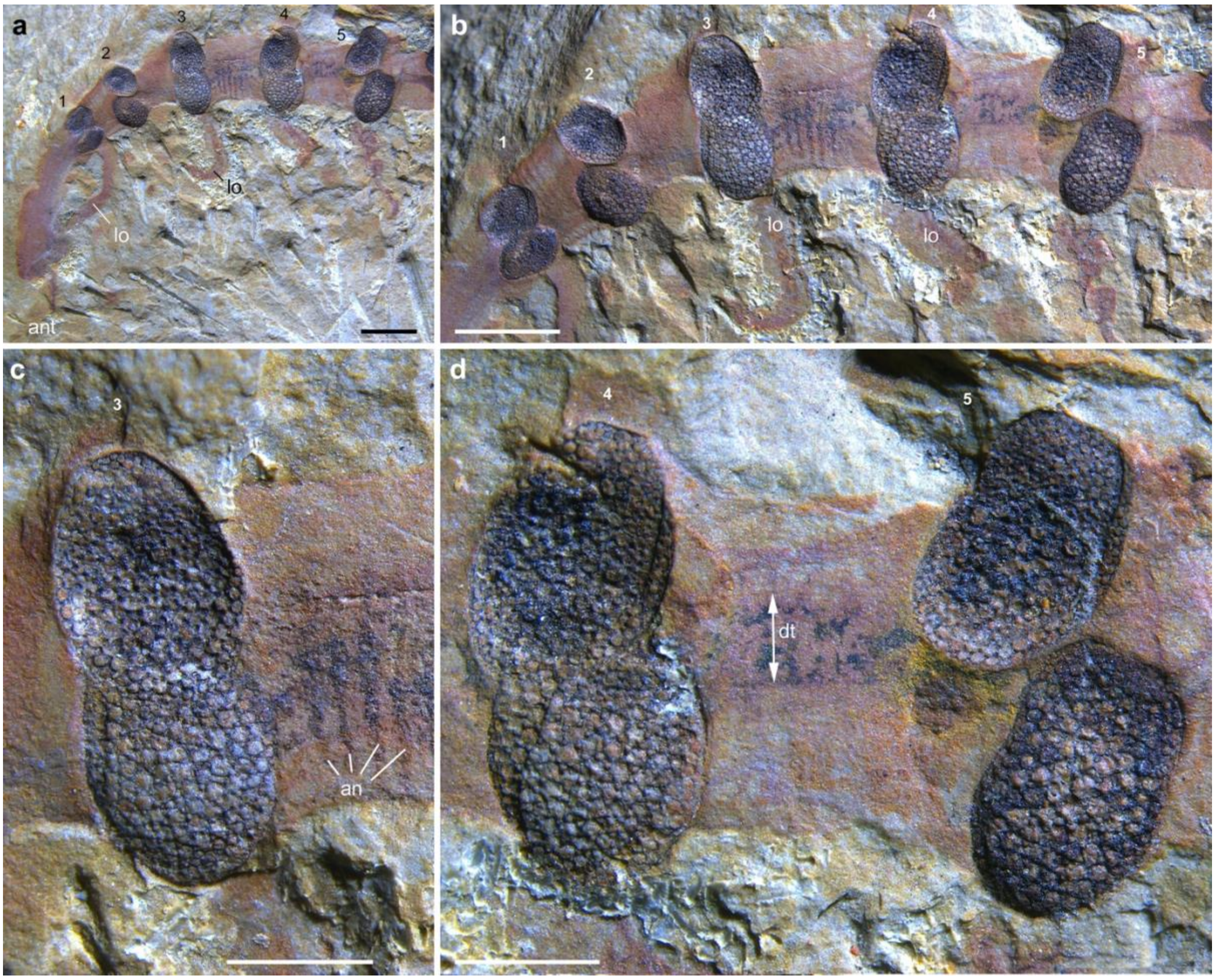

a-d Complete specimen (YM-YD082; Maotianshan section), general view of anterior half and details of duplicated plates. Abbreviations: an, body annulation; ant, anterior end; dt, digestive tract; lo, lobopod (leg); 1-5, 1st-to-5th sclerite. Scale bars: 2 mm in **a, b**; 1 mm in **c, d**.

Supplementary Figure 2. Cuticle renewal in *Microdictyon*: successive steps.

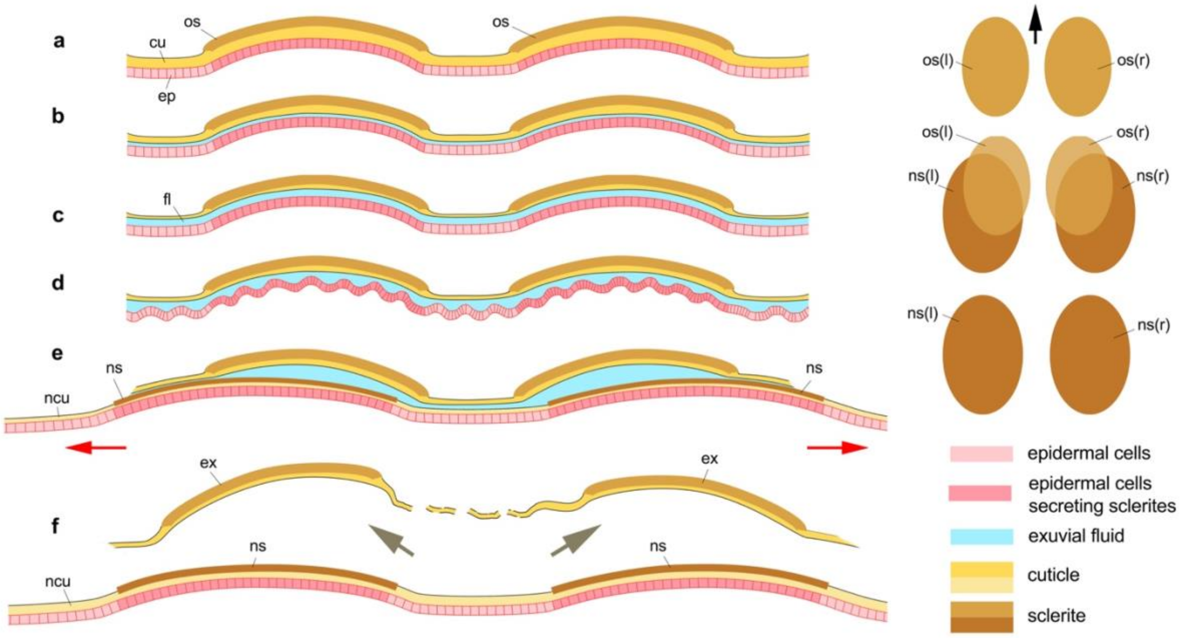

a Intermolt stage showing cuticle and plates secreted by epidermal cells. **b, c** Apolysis; secretion of fluid between epidermis and cuticle; lower part of cuticle lysed. **d** Cell division within epidermis. **e** New cuticle secreted. **f** Ecdysis; old cuticle splits. Right sketches represent the outlines of a pair of sclerites in dorsal view. At step **e**, just before ecdysis, new and old sclerites overlap each other. Red arrows indicate expansion of epidermis and underlying tissues, gray arrows cuticular split. Abbreviations: cu, cuticle; ep: epidermis; fl, fluid; ncu, new cuticle; ns, new sclerite; ns(l), left new sclerite; ns(r), right new sclerite; os, old sclerite; os(l), left old sclerite; os(r), right old sclerite.

Supplementary Figure 3. Assumed molting process in *Microdictyon*.

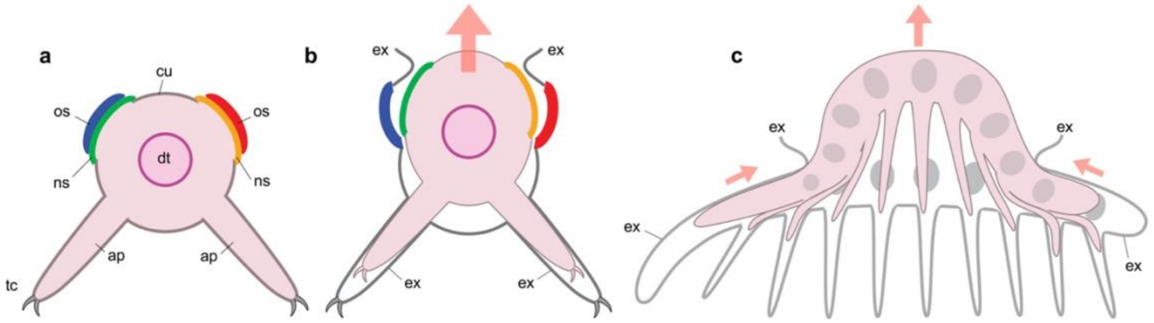

a Before ecdysis; new cuticle has already formed; old and new sclerites overlap each other. **b** Ecdysis; the animal shed its old cuticle including sclerites through a dorsal split. **c** Idealized reconstruction of ecdysis; the animal extricates itself from its old cuticle (exuvia). Light red arrows indicate animal's movement. Abbreviations: ap, appendage; cu, cuticle; dt, digestive tract; ex, exuvia; ns, new sclerite; os, old sclerite; tc, terminal claws. Based on observations of molting from living shrimps, they split dorsal mid-line and extricate anterior end (head) first and then the posterior end.
